# Normative reference values of the phase angle for Korean population: an analysis of the Korea National Health and Nutrition Examination Survey

**DOI:** 10.1007/s40520-025-03130-4

**Published:** 2025-07-19

**Authors:** Daehyun Lee, Chang Won Won, Miji Kim

**Affiliations:** 1 KHU-KIST Department of Converging Science and Technology, Graduate School, Kyung Hee University, Seoul, Republic of Korea; 2 Elderly Frailty Research Center, Department of Family Medicine, College of Medicine, Kyung Hee University, Seoul, Republic of Korea; 3 Department of Health Sciences and Technology, College of Medicine, Kyung Hee University, Seoul, Republic of Korea

**Keywords:** Phase angle, Normative values, Sarcopenia, Korea population, Cutoff point

## Abstract

**Background:**

Phase angle (PhA) reflects fluid balance and cellular membrane integrity within the human body. Establishing normative reference values for PhA is crucial for research and clinical purposes.

**Objectives:**

This study aimed to establish normative reference PhA values in a representative sample of the Korean population and determine the cutoff values for diagnosing sarcopenia in older adults.

**Methods:**

This cross-sectional analysis included the data of 4,881 individuals aged 10–80 years. Multi-frequency bioelectrical impedance analysis was employed for PhA measurements. Sarcopenia was defined according to the Asian Working Group for Sarcopenia 2019 guidelines. The Lambda-Mu-Sigma method was applied to create centile curves and tables. Sensitivity, specificity, and area under the receiver operating characteristic curve were used to determine the sarcopenia cutoff points, and an association between PhA and sarcopenia was assessed using a weighted logistic regression.

**Results:**

PhA values increased during teenage years and peaked in the 30s for both sexes, with a slower decline in women than in men from midlife. PhA was higher in men than in women across most body mass indices and age groups (*p* < 0.05). The PhA cutoff points for diagnosing sarcopenia in older adults were 4.65^°^ and 4.25^°^ for men and women, respectively. A significant association between PhA and sarcopenia was found in both men (odds ratio [OR]: 6.63; 95% confidence interval [CI]: 2.97–14.79) and women (OR: 3.12; 95% CI: 1.50–6.48) after adjusting for confounders.

**Conclusions:**

This study is the first to establish normative reference values for PhA across the lifespan of a Korean population aged 10–80 years and the cutoff points for diagnosing sarcopenia in older adults.

**Supplementary Information:**

The online version contains supplementary material available at 10.1007/s40520-025-03130-4.

## Introduction

Bioelectrical impedance analysis (BIA) is a noninvasive, cost-effective, portable, and efficient method for estimating body composition and health status by measuring resistance using low-voltage alternating currents [1]. Resistance reflects the opposition to electric current flow through body ionic fluids, whereas reactance indicates the delay in current flow produced by capacitive components such as cell membranes and tissue interfaces. Reactance causes the current to lag behind the voltage, resulting in a phase shift known as the phase difference [2]. The phase angle (PhA), which reflects the phase shift, has primarily been utilized in research based on measurements taken at a frequency of 50 kHz. It is calculated using the formula: $\:\text{P}\text{A}\:\left(\text{d}\text{e}\text{g}\text{r}\text{e}\text{e}\text{s}\right)=\text{a}\text{r}\text{c}\text{t}\text{a}\text{n}\left(\frac{\text{R}\text{e}\text{a}\text{c}\text{t}\text{a}\text{n}\text{c}\text{e}}{\text{R}\text{e}\text{s}\text{i}\text{s}\text{t}\text{a}\text{c}\text{n}\text{e}}\right)\times\:\left(\frac{180}{{\uppi\:}}\right)$ [3, 4].

PhA reflects the cell mass contained in soft tissues and is advantageous for assessing cellular health and nutritional status [5, 6]. Therefore, it is a valuable tool for assessing health status and predicting disease severity and survival in both clinical and research settings [1, 7, 8]. Higher PhA values indicate greater cellularity, better cell membrane integrity, and improved cellular function [9]. Conversely, low PhA values are prognostic indicators of poor health outcomes, including acute and chronic inflammation, disease, and mortality [8–14]. Notably, individuals with chronic conditions such as liver cirrhosis, chronic obstructive pulmonary disease, hemodialysis, and cancer exhibit lower PhA values than healthy individuals [8, 15–17].

Establishing reference values for PhA is crucial for assessing whether an individual’s PhA values fall within a normal range. PhA values vary according to population characteristics, including ethnicity, body mass, and physical activity [18, 19]. Although reference PhA values that account for age, sex, and body mass index (BMI) have been published in Germany, Italy, Switzerland, and the United States [19–23], no such reference values have been published for East Asia, including Korea. Consequently, establishing reference values for PhA in the Korean population, considering age, sex, and BMI, is essential.

Sarcopenia, the age-related loss of muscle mass and function, is associated with several adverse health outcomes, including disability, falls, fractures, hospitalization, and mortality [24]. Previous studies have reported an association between decreased PhA and sarcopenia, relating increased sarcopenia prevalence with lower PhA levels [17, 25, 26]. Therefore, exploring the association between PhA and sarcopenia in the Korean population could be significant, particularly in establishing the reference PhA values for the Korean population.

This study aimed to establish the reference values for PhA using a population-representative sample of Korean individuals aged 10–80 years from the Ninth Korea National Health and Nutrition Examination Survey (KNHANES IX, 2022) and explore the association between PhA and sarcopenia based on the cutoff points for sarcopenia using PhA values in older adults.

## Materials and methods

### Study design and population

The KNHANES IX is a nationwide, population-based, cross-sectional health examination survey conducted by the Division of Chronic Disease Surveillance, Korea Disease Control and Prevention Agency (KDCA), Ministry of Health and Welfare. Since 1998, KNHANES has been conducted annually to monitor the health and nutrition status of the South Korean population [27]. Participants are selected each year using stratified, multistage probability sampling by household unit.

KNHANES data are publicly available with having been rigorously reviewed and cross-checked the for missing information, erroneous values, incorrect codes, logical inconsistencies, missing values, and statistical outliers. We then selected 4,881 individuals (2,178 men and 2,703 women) aged ≥ 10 years from the total 6,265 participants in the KNHANES IX after excluding 1,344 and 40 with missing BIA data and height measurements, respectively (Supplementary Fig. 1).

### Measurement of anthropometric parameters

Trained examiners measured the anthropometric parameters. Height was recorded to the nearest 0.1 cm using a wall-mounted stadiometer. Weight was measured to the nearest 0.1 kg, with participants wearing light clothing without shoes. BMI was calculated by dividing weight in kilograms by height in meters squared.

### Sarcopenia diagnosis

Sarcopenia was defined according to the updated guidelines of the Asian Working Group for Sarcopenia 2019 [28]. An appendicular skeletal muscle mass (ASM) index of < 7.0 kg/m² in men and < 5.7 kg/m² in women assessed using BIA was defined as low muscle mass. A handgrip strength of < 28 kg for men and < 18 kg for women was defined as low muscle strength. Notably, KNHANES data only include muscle mass and handgrip strength and do not encompass the criteria for assessing low physical performance.

### Phase angle and appendicular skeletal muscle mass measurement

In the KNHANES IX dataset, a multifrequency BIA (Inbody 970; InBody Co., Ltd., Seoul, Korea), which has been validated as a reliable tool for assessing body composition, was used to measure various parameters, including muscle mass (excluding bone minerals), fat mass, total body water, and whole-body PhA. A recent study involving Korean adults aged 20 to 70 years reported strong correlations between ASM measured by the InBody 970 and two dual-energy X-ray absorptiometry models (Hologic QDR-4500 W and GE-Lunar Prodigy), with correlation coefficients exceeding 0.8 for both devices and surpassing 0.9 for the Hologic model [29, 30]. 

Measurements were performed after removing accessories and any metallic objects from the body, ensuring the participants were on an empty stomach, as fasting is necessary to prevent result alterations. The participants wore light clothing and were barefoot, with their heels aligned with the electrodes. Individuals with pacemakers, with implantable cardioverter-defibrillators, or who were pregnant were excluded from this study.

Regional measurements of muscle and fat masses were obtained for the right arm, left arm, right leg, and left leg. The ASM was calculated by summing the muscle masses of both arms and legs, and the ASM index was calculated by dividing the ASM (kg) by the height (m²). Whole-body PhA was measured on the right side of the body, including the right arm, torso, and right leg at 50 kHz.

### Measurement of muscle strength

Handgrip strength was assessed using a digital handgrip dynamometer (T.K.K.5401; Takei Scientific Instruments Co., Ltd., Tokyo, Japan). Individuals who had undergone hand or wrist surgery in the last 3 months; were unable to hold the dynamometer with either hand (e.g., due to paralysis or missing limbs); or experienced pain, aching, or stiffness in their right hand in the past 7 days (e.g., arthritis, tendonitis, and carpal tunnel syndrome) were excluded. The participants stood upright with their shoulders in a neutral position, elbows fully extended, and arms at their sides. A trained examiner provided instructions, and the dynamometer handles were adjusted to fit each participant’s hand size. The participants then squeezed the grip handle with maximum force for 3 s, first for a practice trial, followed by two measured attempts. Handgrip strength was measured twice for each hand, and the maximum handgrip strength was recorded as the highest value obtained for each hand.

### Ethics

The KNHANES was approved by the Institutional Review Board (IRB) of the KDCA, and all participants provided written informed consent (IRB number: 2018-01-03–4 C-A). This study was exempted from review by the IRB of Kyung Hee University (IRB number 2024-07-039).

### Statistical analysis

The KNHANES IX (2022) utilized a two-stage stratified cluster sampling method to represent the Korean population. Considering the complex survey design involving stratified, random, and cluster sampling, we applied sample weights based on the methods recommended by the KDCA [27]. The means and standard errors of PhA were tabulated for each sex across the 5-year age groups, BMI groups, and combined 10-year age and BMI groups.

Weighted descriptive statistics was employed in complex sampling analysis to estimate the mean PhA values across the entire distribution (0–100%) and within groups defined by sex, age, and BMI. Furthermore, Youden’s index, along with the sensitivity, specificity, and area under the receiver operating characteristic curve (AUC) analyses, was used to determine the PhA cutoff points for diagnosing sarcopenia. Weighted logistic regression was conducted to examine the association between PhA cutoff points and sarcopenia, and the results were presented as odds ratios (ORs) and 95% confidence intervals (CIs). The final model was adjusted for age and BMI. Statistical significance was defined as a two-tailed p-value of < 0.05. All statistical analyses were performed using SAS version 9.3 (SAS Institute Inc., Cary, NC, USA) and IBM SPSS Statistics 28.0 package (SPSS Inc., Chicago, IL, USA).

Sex- and age-specific percentile curves for PhA were generated using the Lambda-Mu-Sigma (LMS) method implemented within the GAMLSS package in R (version 4.3.2). Predicted centile values, ranging from the 1st to the 99th percentiles, were estimated across age 10 to 80 years, grouped into 5-year intervals. The LMS approach assumes that the outcome variable follows a normal distribution after a Box-Cox transformation, with the distribution at each age described by three parameters: lambda (λ, skewness), mu (µ, median) and sigma (σ, coefficient of variation). In addition, empirically weighted percentiles were calculated to evaluate PhA values by sex, age, and body mass index [31, 32]. 

## Results

The mean age of men was higher than that of the women in the study population (44.40 ± 0.62 years vs. 46.23 ± 0.54 years, *p* < 0.01). The mean BMI and ASM were also higher in men than in women (24.76 ± 0.62 kg/m^2^ vs. 23.13 ± 0.11 kg/m^2^; 23.21 ± 0.12 kg vs. 15.56 ± 0.06 kg, all *p* < 0.01). The body fat percentage was lower in men than in women (24.58 ± 0.17% vs. 32.45 ± 0.17%, *p* < 0.01) (Table 1).

**Table 1:** Characteristics of the study participants (*n* = 4,881) weighted mean (standard error)

Variables	Men (*n* = 2,178)	Women (*n* = 2,703)	*p*-value
Age (years)	44.40 (0.62)	46.23 (0.54)	< 0.01
Body weight (kg)	73.05 (0.37)	58.34 (0.28)	< 0.01
Height (cm)	171.41 (0.22)	158.82 (0.18)	< 0.01
Body mass index (kg/m^2^)	24.76 (0.10)	23.13 (0.11)	< 0.01
Appendicular muscle mass (kg)	23.21 (0.12)	15.56 (0.06)	< 0.01
Fat mass (kg)	18.39 (0.18)	19.37 (0.19)	< 0.01
Body fat percentage (%)	24.58 (0.17)	32.45 (0.17)	< 0.01
Handgrip strength (kg)	39.93 (0.31)	24.34 (0.14)	< 0.01
Extracellular water (L)	15.15 (0.07)	19.37 (0.19)	< 0.01
Intracellular water (L)	25.01 (0.12)	32.45 (0.17)	< 0.01

**Fig. 1:**
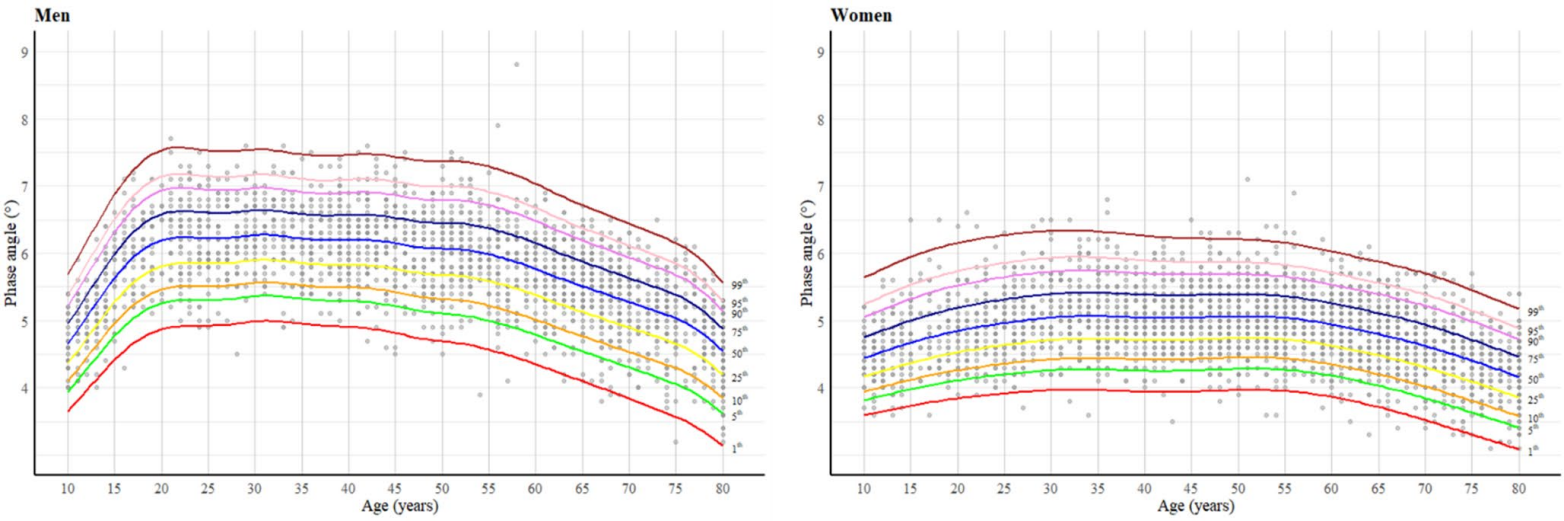
Phase angle percentile curves for men (left) and women (right)

Figure 1; Table 2 show the percentile curves and scatter plots of PhA for men and women aged 10–80 years in the Korean population. In men, PhA increases steeply during 10s, followed by a relatively stable plateau from their 20s to the 50s. A gradual decline begins in the mid-50s with a marked acceleration observed in the mid-70s. Meanwhile, in women, although PhA also increases during 10s, the rate of increase is more gradual compared to men. From their 20s to 50s, women maintain a relatively stable plateau, with a noticeable decline observed beginning in their mid-60s. Supplementary Fig. 2 presents the percentile PhA values stratified by sex and 10-year age intervals. Notably, in both men and women, the highest PhA values were observed in their 30s, while the lowest values were observed in the 70–80-year age group.

**Table 2:** Weighted mean, percentiles, and unweighted mean for phase angle according to sex and age groups (*n* = 4,881)

Sex	Age (years)	*n*	Weighted						Unweighted	
			Mean ± SE	Percentile					Mean ± SD	
				20th	40th	50th	60th	80th		
Men	10–14	145	5.01 ± 0.04	4.57	4.86	4.99	5.12	5.43	5.00 ± 0.52	
	15–19	97	5.96 ± 0.06	5.47	5.83	5.98	6.12	6.46	5.96 ± 0.58	
	20–24	114	6.22 ± 0.06	5.72	6.07	6.23	6.38	6.73	6.21 ± 0.62	
	25–29	121	6.21 ± 0.05	5.74	6.07	6.21	6.35	6.66	6.19 ± 0.56	
	30–34	113	6.33 ± 0.05	5.90	6.22	6.35	6.48	6.76	6.30 ± 0.53	
	35–39	145	6.22 ± 0.05	5.72	6.01	6.14	6.27	6.60	6.17 ± 0.54	
	40–44	163	6.19 ± 0.05	5.73	6.07	6.21	6.35	6.66	6.19 ± 0.55	
	45–49	146	6.07 ± 0.05	5.58	5.92	6.06	6.20	6.53	6.04 ± 0.58	
	50–54	175	6.05 ± 0.06	5.60	5.96	6.10	6.25	6.58	6.06 ± 0.57	
	55–59	162	5.92 ± 0.07	5.38	5.70	5.85	6.00	6.37	5.87 ± 0.59	
	60–64	194	5.63 ± 0.04	5.22	5.53	5.67	5.79	6.08	5.64 ± 0.52	
	65–69	222	5.38 ± 0.06	4.98	5.33	5.47	5.60	5.87	5.42 ± 0.57	
	70–74	162	5.06 ± 0.07	4.65	5.00	5.14	5.27	5.58	5.11 ± 0.57	
	75–79	133	4.93 ± 0.05	4.48	4.80	4.93	5.07	5.38	4.93 ± 0.57	
	80	86	4.48 ± 0.05	4.02	4.34	4.47	4.59	4.87	4.45 ± 0.52	
Women	10–14	127	4.51 ± 0.04	4.14	4.38	4.48	4.59	4.85	4.51 ± 0.43	
	15–19	87	4.85 ± 0.06	4.43	4.69	4.81	4.94	5.25	4.85 ± 0.53	
	20–24	132	4.94 ± 0.05	4.49	4.75	4.88	5.01	5.33	4.88 ± 0.51	
	25–29	141	5.02 ± 0.04	4.57	4.85	4.97	5.10	5.41	5.01 ± 0.51	
	30–34	144	5.09 ± 0.05	4.68	4.97	5.10	5.23	5.52	5.14 ± 0.53	
	35–39	177	5.08 ± 0.05	4.66	4.92	5.04	5.17	5.49	5.08 ± 0.50	
	40–44	229	5.00 ± 0.03	4.56	4.87	4.99	5.12	5.39	4.99 ± 0.47	
	45–49	224	5.07 ± 0.04	4.69	4.95	5.06	5.18	5.46	5.06 ± 0.46	
	50–54	254	5.07 ± 0.03	4.66	4.94	5.06	5.18	5.46	5.06 ± 0.49	
	55–59	203	5.03 ± 0.05	4.60	4.87	4.99	5.11	5.42	4.99 ± 0.47	
	60–64	295	4.88 ± 0.03	4.51	4.78	4.90	5.01	5.26	4.90 ± 0.46	
	65–69	249	4.75 ± 0.03	4.39	4.65	4.77	4.88	5.13	4.76 ± 0.45	
	70–74	186	4.53 ± 0.04	4.08	4.39	4.53	4.66	4.98	4.53 ± 0.52	
	75–79	153	4.32 ± 0.04	3.95	4.20	4.31	4.42	4.67	4.30 ± 0.44	
	80	102	4.12 ± 0.06	3.77	4.04	4.15	4.27	4.54	4.17 ± 0.46	

SD, standard deviation; SE, standard error

Supplementary Table 1 displays the mean PhA values according to BMI groups separated by sex. For men, mean PhA values consistently increased from a BMI of < 18.5 to ≥ 35.0 kg/m^2^ (*p* for trend < 0.01). For women, the mean PhA values increased consistently up to a BMI of < 35.0 (*p* for trend < 0.01); however, the mean PhA values for a BMI of ≥ 35.0 kg/m^2^ remained stable, similar to those for ≤ 30.0 to < 35.0 kg/m^2^. Supplementary Table 2 presents detailed PhA values according to BMI, sex, and age groups. For both sexes, the mean PhA values gradually increased with age across all BMI groups. The PhA values were generally higher in men than in women across most BMI and age groups (all, *p* < 0.05), with exceptions for individuals with BMI < 18.5 kg/m^2^ in their 50s and 70s and older, ≤ 30.0 to < 35.0 kg/m^2^ in the teenage years, and ≥ 35.0 kg/m^2^ in their 60s.

The characteristics of older adults are presented in Supplementary Table 3 The prevalence rates of sarcopenia in men and women were 7.7% and 10.5%, respectively. Table 3 displays the PhA cutoff points for identifying sarcopenia, with the corresponding AUC values presented in Supplementary Fig. 3 For men, the PhA cutoff point for sarcopenia was ≤ 4.65^°^, with sensitivity, specificity, and AUC of 73.52%, 79.66%, and 0.811 (95% CI: 0.810–0.812), respectively. For women, the cutoff point was ≤ 4.25^°^, with a sensitivity, specificity, and AUC of 68.82%, 74.45%, and 0.785 (95% CI: 0.784–0.785), respectively.

**Table 3:** Phase angle cutoff points for diagnosing sarcopenia in older adults

	Men		Women	
Phase angle values	Sensitivity	Specificity	Sensitivity	Specificity
≤ 3.75°	18.68%	98.49%	30.94%	95.81%
≤ 3.85°	20.36%	97.78%	34.49%	92.16%
≤ 3.95°	28.65%	97.49%	40.54%	89.52%
≤ 4.05°	28.65%	95.53%	48.68%	85.68%
≤ 4.15°	32.63%	94.58%	56.27%	79.65%
**≤ 4.25**°	41.96%	91.49%	**68.82%**	**74.45%**
≤ 4.35°	48.68%	89.13%	72.18%	65.41%
≤ 4.45°	52.80%	86.51%	79.52%	58.88%
≤ 4.55°	65.32%	83.10%	89.32%	51.28%
**≤ 4.65**°	**73.52%**	**79.66%**	89.32%	42.40%
≤ 4.75°	73.52%	75.69%	95.15%	32.97%
≤ 4.85°	75.44%	70.36%	97.78%	26.17%

Phase angle cutoff points are in bold

Table 4 shows the association between low PhA and sarcopenia in older adults after adjusting for age and BMI. In both men and women, a significant association between PhA and sarcopenia was observed in both unadjusted (OR: 10.87; 95% CI: 5.27–22.42 for men; OR: 6.63; 95% CI: 2.97–14.79 for women) and adjusted models (OR: 6.43; 95% CI: 3.40–12.20 for men; OR: 3.12; 95% CI: 1.50–6.48 for women).

**Table 4:** Association between phase angle and sarcopenia

	Men (*n* = 548)					
	OR (95% CI)		*p*-value, crude		Adjusted OR (95% CI)	*p*-value, adjusted
Phase angle						
≥ 4.65	*Ref.*				*Ref.*	
< 4.65	10.87 (5.27–22.42)		*p* < 0.001		6.63 (2.97–14.79)	*p* < 0.001
	Women (*n* = 578)					
	OR (95% CI)		*p*-value, crude		Adjusted OR (95% CI)	*p*-value, adjusted
Phase angle						
≥ 4.25	*Ref.*				*Ref.*	
< 4.25	6.43 (3.40–12.20)		*p* < 0.001		3.12 (1.50–6.48)	*p* = 0.0025

Survey logistic regression analysis adjusted for age and BMI

BMI, body mass index; CI, confidence intervals; OR, odds ratio

## Discussion

This study determined the reference PhA values in a Korean population. To the best of our knowledge, this is the first study to examine the PhA percentiles and reference values in a population from Eastern countries. Our findings indicated that the PhA values increase until the 30s and then start to decline in both sexes. Moreover, men exhibited higher PhA values compared with women, and individuals with higher BMI levels exhibited higher PhA values than those with lower BMI levels. We also identified the PhA cutoff points for diagnosing sarcopenia in individuals aged ≥ 65 years.

PhA values increased from the teenage years until the 30s in both men (6.33 ± 0.05°) and women (5.09 ± 0.05°), decreased smoothly in their mid-40s and declined more rapidly from the mid-50s onward. A systematic review, primarily focused on Western countries, reported that the mean PhA values progressively increased from early childhood until the age of 18 years, stabilized in midlife at approximately 7.3^°^ for men and 6.4^°^ for women, and then decreased after the age of 48 years [23]. Similarly, a recent study in Italy reported that the PhA starts decreasing in the mid-30s, with a more pronounced decline from the 50s onward in both sexes [21]. Although the specific age at which the PhA value begins to decrease may vary due to differences in population characteristics and measurement techniques used, the overall trend of decreasing PhA values with aging is consistent across studies [23].

We also observed an increase in PhA with BMI, which is consistent with the results of previous studies [19, 20]. However, this trend in mean PhA values was not consistent in women with a BMI of ≥ 35.0 kg/m^2^. Notably, the higher BMI groups exhibited a gradual increase in PhA in both sexes, likely due to greater cell mass. This anomaly in women might be attributed to increased tissue hydration, leading to a higher proportion of extracellular water within total body water, or a pathophysiological fluid overload from increased soft tissue mass despite normal tissue hydration in severe obesity [33, 34]. Additionally, the decrease in PhA might be influenced by severe obesity associated with cellular membrane damage due to chronic inflammation [12]. Studies in the German population and a previous systematic review have also reported an increase in PhA up to a BMI of 35.0 kg/m^2^, followed by a subsequent decrease [20]. This finding aligns with observations that women with BMIs of 40–60 kg/m² had a higher prevalence of low PhA than those with BMIs of 30–35 kg/m² [35].

Consistent with the results of previous studies, our study revealed higher PhA levels in men than in women [6, 20], which may be attributed to higher muscle mass and a lower body fat percentage in men than women [36, 37]. Hormonal differences may considerably cause differences in body composition, and higher testosterone levels in men can enhance cell membrane stability and muscle function more effectively than estrogen [38, 39]. Although both testosterone and estrogen protect the skeletal muscle, testosterone’s role in muscle generation is more well-documented. This difference in body composition, specifically the higher water and electrolyte content in the muscle tissue, contributes to higher electrical conductivity, resulting in higher PhA in men [40, 41]. Furthermore, PhA determinants may vary by sex, with age and stature exhibiting more significant impact in men and extracellular water-to-intracellular water ratio and fat-free mass, measured via underwater weighting, exhibiting more impact in women [42].

Our findings revealed an association between low PhA and sarcopenia in older adults after adjusting for age and BMI. PhA values are inversely related to sarcopenia in older adults and may serve as reliable bioelectrical markers for identifying sarcopenia risk [6, 43]. A study conducted in Poland identified PhA cutoff points as 5.42^°^ for men and 4.76^°^ for women for pre-sarcopenia in a population aged ≥ 50 years according to the European Working Group on Sarcopenia in Older People 2 criteria, revealing a significant association between lower PhA and sarcopenia compared with individuals without sarcopenia. Similarly, a recent study in Mexico reported a PhA cutoff point of 4.1^°^ for both sarcopenia and frailty in adults aged ≥ 50 years, demonstrating an association between low PhA values and sarcopenia (OR: 8.4; 95% CI: 3.85–18.4) [44, 45]. In addition, recent evidence shows a correlation between PhA and sarcopenia diagnostic components in older men with cancer, with the mean PhA being lower in those with sarcopenia (5.02^°^ vs. 4.18^°^, *p* < 0.01) [46]. The association between PhA values and sarcopenia indicates that PhA may not only reflect body composition, including muscle mass, fat mass, and body fluid or nutritional status, but also serve as an indicator of general physical function and health [47–49].

This study has some limitations. First, the cross-sectional design cannot establish causality of the relationship. Second, our study included a Korean population aged 10–80 years without excluding individuals with diseases such as liver cirrhosis, chronic obstructive pulmonary disease, hemodialysis, and cancer. Lastly, although we used a representative dataset of the Korean population, the identified cutoff points may not fully represent the national context. Considering the lack of prior studies in this population, further research is warranted to validate the PhA cutoff points for diagnosing sarcopenia and ensure external validity.

## Conclusions

Our findings revealed a progressive increase in PhA values from the age of 10 to 30 years in both sexes in the Korean population. Additionally, we identified cutoff points for PhA values and their association with sarcopenia. Further research is needed to compare the PhA values and cutoff points across different populations to establish nationally representative values.

## Electronic supplementary material

Below is the link to the electronic supplementary material.

Supplementary Material 1

## Data Availability

The data described in the manuscript, codebook, and analytical code will be made publicly available at KNHANES website (http://knhanes.cdc.go.kr) without restriction.
