# Supplementary material for: Normative reference values of the phase angle for Korean population: an analysis of the Korea National Health and Nutrition Examination Survey
Source: Aging Clin Exp Res. 2025 Jul 19;37(1):226. doi: 10.1007/s40520-025-03130-4 (PMC12276114; doi:10.1007/s40520-025-03130-4)
Supplement: Supplementary file 1 — Supplementary Material 1 [file 40520_2025_3130_MOESM1_ESM.docx]

**Supplementary Information**

**Title:** Normative Reference Values of the Phase Angle for Korean Population: An Analysis of the Korea National Health and Nutrition Examination Survey

Daehyun Lee^a^, Chang Won Won^b*^, and Miji Kim^c*^

^a^ KHU-KIST Department of Converging Science and Technology, Graduate School, Kyung Hee University, Seoul, 02447, Republic of Korea

E-mail: lyh737@naver.com

^b^ Elderly Frailty Research Center, Department of Family Medicine, College of Medicine, Kyung Hee University, Seoul, 02447, Republic of Korea

E-mail: [chunwon@khmc.or.kr](mailto:chunwon@khmc.or.kr)

^c^ Department of Health Sciences and Technology, College of Medicine, Kyung Hee University, Seoul, 02447, Republic of Korea

E-mail: [mijiak@khu.ac.kr](mailto:mijiak@khu.ac.kr)

*Correspondence: These authors equally contributed to this work as co-corresponding authors


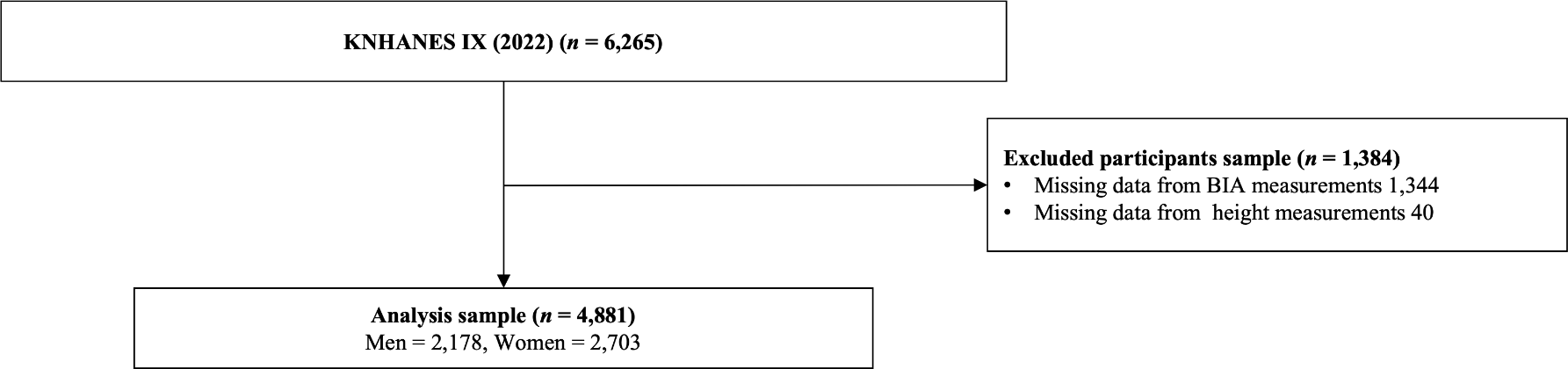


**Supplementary Fig. 1** Flowchart of the study population

*
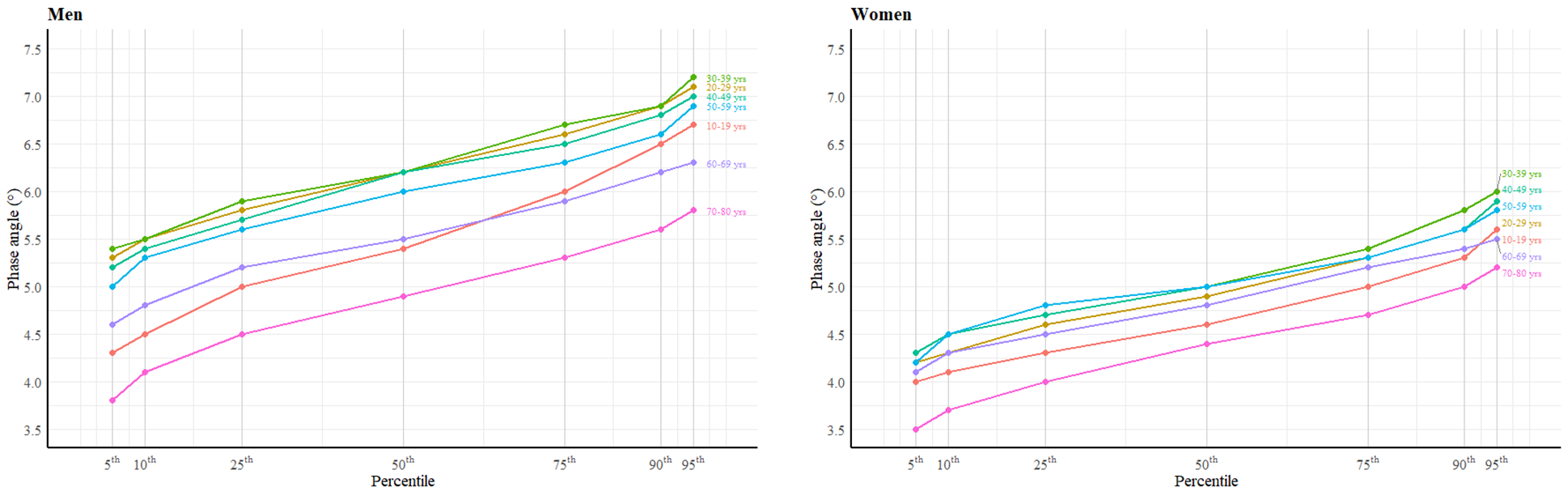
*

**Supplementary Fig. 2** Weighted percentiles of phase angle by sex and age (grouped into 10-year intervals)


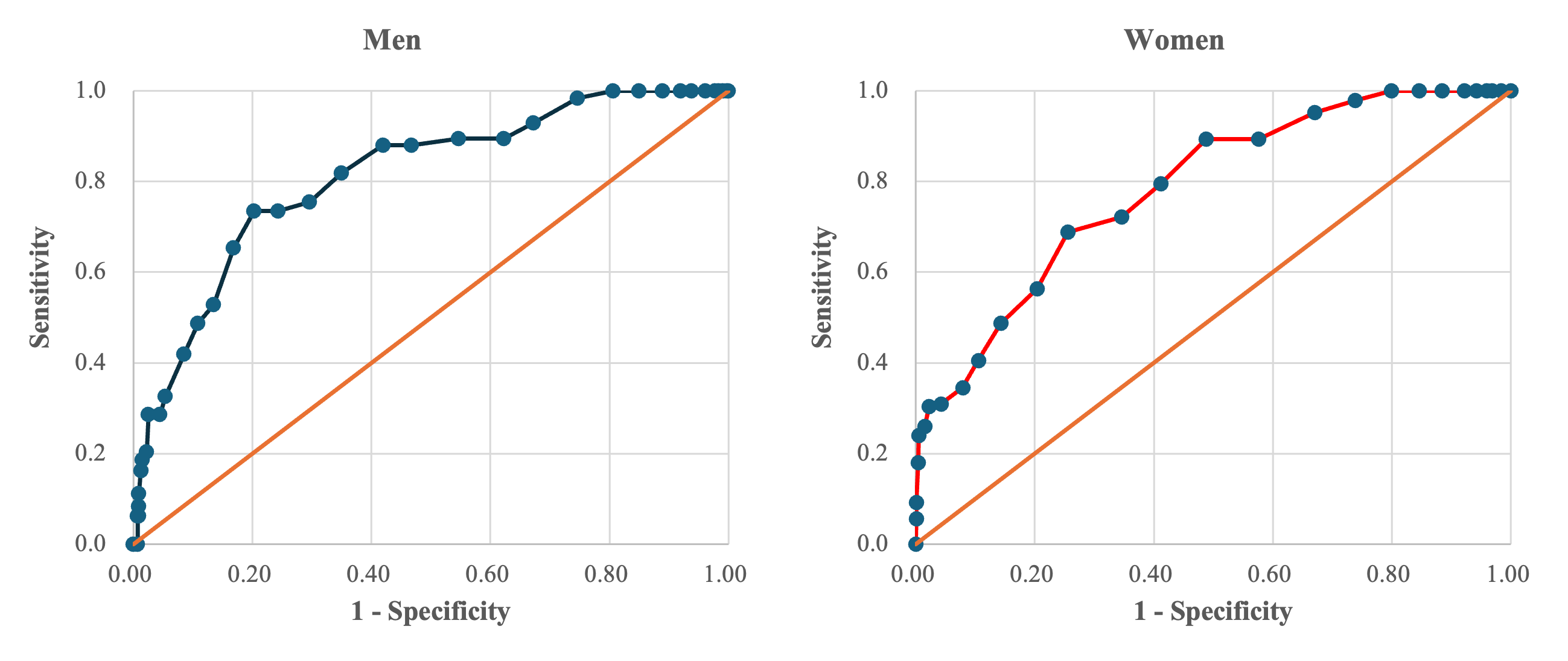


**Supplementary Fig. 3** Receiver operating characteristic curve for men (area under curve = 0.811) and women (area under curve = 0.785)

**Supplementary Table 1** Mean, standard error, and percentiles for phase angle according to sex and body mass index groups (*n* = 4,881)

| Sex | BMI (kg/m^2^) | *n* | Weighted | | | | | | Unweighted |
| --- | --- | --- | --- | --- | --- | --- | --- | --- | --- |
|  |  |  | Mean ± SE | Percentile | | | | | Mean ± SD |
|  |  |  |  | 20^th^ | 40^th^ | 50^th^ | 60^th^ | 80^th^ |  |
| Men | <18.5 | 93 | 4.99 ± 0.10 | 4.24 | 4.84 | 5.00 | 5.16 | 5.63 | 4.89 ± 0.71 |
|  | ≤18.5 ≤ to <25.0 | 1,202 | 5.73 ± 0.03 | 5.12 | 5.54 | 5.72 | 5.90 | 6.28 | 5.58 ± 0.73 |
|  | ≤25.0 to <30.0 | 723 | 6.06 ± 0.03 | 5.49 | 5.92 | 6.07 | 6.20 | 6.55 | 5.91 ± 0.70 |
|  | ≤30.0 to <35.0 | 133 | 6.31 ± 0.08 | 5.73 | 6.12 | 6.25 | 6.43 | 6.79 | 6.16 ± 0.74 |
|  | ≥35.0 | 27 | 6.36 ± 0.12 | 5.68 | 6.18 | 6.49 | 6.62 | 6.81 | 6.32 ± 0.60 |
| Women | <18.5 | 218 | 4.49 ± 0.03 | 4.04 | 4.29 | 4.43 | 4.57 | 4.88 | 4.46 ± 0.47 |
|  | ≤18.5 to <25.0 | 1,709 | 4.86 ± 0.01 | 4.41 | 4.69 | 4.81 | 4.93 | 5.23 | 4.83 ± 0.51 |
|  | ≤25.0 to <30.0 | 623 | 5.03 ± 0.03 | 4.49 | 4.85 | 4.98 | 5.11 | 5.43 | 4.97 ± 0.57 |
|  | ≤30.0 to <35.0 | 124 | 5.26 ± 0.06 | 4.81 | 5.08 | 5.19 | 5.37 | 5.65 | 5.26 ± 0.60 |
|  | ≥35.0 | 29 | 5.26 ± 0.09 | 4.88 | 5.11 | 5.21 | 5.29 | 5.65 | 5.21 ± 0.53 |

SD, standard deviation; SE, standard error

**Supplementary Table 2** Phase angle of the Korean population according to sex, body mass index, and age group

|  | Men (*n* = 2,178) | | | | | | |  | Women (*n* = 2,703) | | | | | | |  | *p*-value |
| --- | --- | --- | --- | --- | --- | --- | --- | --- | --- | --- | --- | --- | --- | --- | --- | --- | --- |
| BMI (kg/m^2^) | Age (years) | n | Mean | ± | SE | 5^th^ | 10^th^ |  | Age (years) | n | Mean | ± | SE | 5^th^ | 10^th^ |  |  |
| <18.5 | 10–19 | 46 | 4.87 | ± | 0.09 | 4.00 | 4.05 |  | 10–19 | 86 | 4.40 | ± | 0.06 | 3.65 | 3.86 |  | <0.01 |
|  | 20–29 | 12 | 5.56 | ± | 0.13 | 4.90 | 4.90 |  | 20–29 | 41 | 4.67 | ± | 0.07 | 3.93 | 4.03 |  | <0.01 |
|  | 30–39 | 7 | 5.57 | ± | 0.10 | 4.90 | 4.94 |  | 30–39 | 25 | 4.74 | ± | 0.07 | 4.01 | 4.22 |  | <0.01 |
|  | 40–49 | 2 | 5.10 | ± | 0.00 | 5.10 | 5.10 |  | 40–49 | 29 | 4.52 | ± | 0.10 | 3.10 | 3.91 |  | <0.01 |
|  | 50–59 | 5 | 4.58 | ± | 0.37 | 3.90 | 3.90 |  | 50–59 | 11 | 4.45 | ± | 0.07 | 3.63 | 3.71 |  | 0.76 |
|  | 60–69 | 7 | 4.86 | ± | 0.26 | 3.90 | 3.90 |  | 60–69 | 12 | 4.20 | ± | 0.18 | 3.30 | 3.30 |  | <0.05 |
|  | ≥70 | 14 | 4.08 | ± | 0.23 | 2.90 | 2.90 |  | ≥70 | 14 | 4.01 | ± | 0.11 | 3.30 | 3.30 |  | 0.77 |
| ≤18.5 to <25.0 | 10–19 | 149 | 5.53 | ± | 0.06 | 4.38 | 4.63 |  | 10–19 | 106 | 4.78 | ± | 0.05 | 4.10 | 4.18 |  | <0.01 |
|  | 20–29 | 126 | 6.17 | ± | 0.05 | 5.21 | 5.52 |  | 20–29 | 180 | 4.93 | ± | 0.04 | 4.16 | 4.30 |  | <0.01 |
|  | 30–39 | 119 | 6.07 | ± | 0.05 | 5.08 | 5.35 |  | 30–39 | 220 | 5.00 | ± | 0.04 | 4.20 | 4.36 |  | <0.01 |
|  | 40–49 | 135 | 5.97 | ± | 0.05 | 5.05 | 5.22 |  | 40–49 | 295 | 5.00 | ± | 0.03 | 4.27 | 4.42 |  | <0.01 |
|  | 50–59 | 169 | 5.93 | ± | 0.05 | 4.97 | 5.21 |  | 50–59 | 308 | 5.00 | ± | 0.03 | 4.20 | 4.35 |  | <0.01 |
|  | 60–69 | 254 | 5.46 | ± | 0.05 | 4.44 | 4.60 |  | 60–69 | 339 | 4.80 | ± | 0.02 | 4.07 | 4.21 |  | <0.01 |
|  | ≥70 | 250 | 4.86 | ± | 0.04 | 3.91 | 4.12 |  | ≥70 | 261 | 4.27 | ± | 0.04 | 3.34 | 3.62 |  | <0.01 |
| ≤25.0 to <30.0 | 10–19 | 34 | 5.75 | ± | 0.10 | 4.78 | 4.93 |  | 10–19 | 20 | 5.23 | ± | 0.10 | 4.33 | 4.42 |  | <0.01 |
|  | 20–29 | 67 | 6.26 | ± | 0.06 | 5.41 | 5.50 |  | 20–29 | 36 | 5.39 | ± | 0.11 | 4.47 | 4.65 |  | <0.01 |
|  | 30–39 | 98 | 6.46 | ± | 0.04 | 5.71 | 5.85 |  | 30–39 | 52 | 5.43 | ± | 0.07 | 4.70 | 4.86 |  | <0.01 |
|  | 40–49 | 136 | 6.27 | ± | 0.05 | 5.40 | 5.61 |  | 40–49 | 97 | 5.21 | ± | 0.05 | 4.34 | 4.61 |  | <0.01 |
|  | 50–59 | 137 | 6.06 | ± | 0.05 | 5.12 | 5.35 |  | 50–59 | 112 | 5.21 | ± | 0.06 | 4.40 | 4.62 |  | <0.01 |
|  | 60–69 | 143 | 5.65 | ± | 0.05 | 4.84 | 4.97 |  | 60–69 | 160 | 4.90 | ± | 0.04 | 4.02 | 4.27 |  | <0.01 |
|  | ≥70 | 108 | 5.06 | ± | 0.07 | 3.92 | 4.17 |  | ≥70 | 146 | 4.47 | ± | 0.04 | 3.70 | 3.92 |  | <0.01 |
| ≤30.0 to <35.0 | 10–19 | 10 | 5.79 | ± | 0.28 | 4.50 | 4.50 |  | 10–19 | 1 | 5.30 | ± | 0.00 | 5.30 | 5.30 |  | 0.08 |
|  | 20–29 | 21 | 6.67 | ± | 0.16 | 5.32 | 5.61 |  | 20–29 | 9 | 5.66 | ± | 0.12 | 5.10 | 5.10 |  | <0.01 |
|  | 30–39 | 30 | 6.44 | ± | 0.09 | 5.72 | 5.81 |  | 30–39 | 17 | 5.70 | ± | 0.14 | 4.80 | 4.93 |  | <0.01 |
|  | 40–49 | 31 | 6.25 | ± | 0.10 | 5.05 | 5.41 |  | 40–49 | 25 | 5.46 | ± | 0.08 | 4.83 | 4.93 |  | <0.01 |
|  | 50–59 | 21 | 6.39 | ± | 0.26 | 5.25 | 5.34 |  | 50–59 | 24 | 5.31 | ± | 0.13 | 4.60 | 4.65 |  | <0.01 |
|  | 60–69 | 11 | 5.59 | ± | 0.21 | 4.70 | 4.72 |  | 60–69 | 28 | 4.98 | ± | 0.07 | 4.14 | 4.42 |  | <0.01 |
|  | ≥70 | 9 | 5.12 | ± | 0.21 | 4.20 | 4.22 |  | ≥70 | 20 | 4.59 | ± | 0.15 | 3.60 | 3.64 |  | <0.05 |
| ≥35.0 | 10–19 | 3 | 6.36 | ± | 0.53 | 5.40 | 5.40 |  | 10–19 | 1 | 4.10 | ± | 0.00 | 4.10 | 4.10 |  | <0.01 |
|  | 20–29 | 9 | 6.43 | ± | 0.23 | 5.30 | 5.30 |  | 20–29 | 7 | 5.28 | ± | 0.11 | 4.80 | 4.81 |  | <0.01 |
|  | 30–39 | 4 | 6.34 | ± | 0.15 | 5.70 | 5.70 |  | 30–39 | 7 | 5.39 | ± | 0.15 | 4.90 | 4.90 |  | <0.01 |
|  | 40–49 | 5 | 6.38 | ± | 0.17 | 5.80 | 5.80 |  | 40–49 | 7 | 5.25 | ± | 0.20 | 4.00 | 4.00 |  | <0.01 |
|  | 50–59 | 5 | 6.18 | ± | 0.24 | 5.50 | 5.50 |  | 50–59 | 2 | 5.09 | ± | 0.11 | 4.80 | 4.80 |  | <0.01 |
|  | 60–69 | 1 | 5.40 | ± | 0.00 | 5.40 | 5.40 |  | 60–69 | 5 | 5.39 | ± | 0.30 | 4.30 | 4.30 |  | 0.98 |
|  | ≥70 | 0 | - | ± | - | - | - |  | ≥70 | 0 | - | ± | - | - | - |  | - |

Weighted mean ± standard error (SE)

**Supplementary Table 3** Characteristics of participants aged ≥65 years (*n* = 1,240)

| Variables | Men (*n* = 594) | Women (*n* = 646) | *p*-value |
| --- | --- | --- | --- |
|  |  |  |  |
| Age (years) | 72.44 ± 5.10 | 72.20 ± 5.03 | 0.40 |
| Weight (kg) | 66.56 ± 9.13 | 57.19 ± 8.18 | <0.01 |
| Height (cm) | 166.78 ± 5.54 | 153.37 ± 5.47 | <0.01 |
| Waist circumference (cm) | 88.57 ± 8.59 | 84.85 ± 9.00 | <0.01 |
| Body mass index (kg/m^2^) | 23.85 ± 2.96 | 24.29 ± 3.36 | <0.05 |
| Appendicular muscle mass (kg) | 20.60 ± 2.85 | 14.21 ± 2.14 | <0.01 |
| Fat mass (kg) | 17.33 ± 5.51 | 20.21 ± 5.77 | <0.01 |
| Body fat percentage (%) | 25.65 ± 5.48 | 34.77 ± 5.79 | <0.01 |
| Handgrip strength (kg) | 34.31 ± 6.58 | 21.72 ± 4.49 | <0.01 |
| Extracellular water | 14.02 ± 1.67 | 10.64 ± 1.22 | <0.01 |
| Intracellular water | 22.20 ± 3.14 | 16.58 ± 1.95 | <0.01 |
| Sarcopenia (*n*) | 46 (7.7) | 68 (10.5) | 0.10 |

Continuous variables are presented as the unweighted mean ± standard deviation; categorical variables are presented as *n* (%)

**Supplementary Table 4** Unweighted mean and standard deviation of the phase angle in older adults by sex and age (*n* = 1,240)

| Variables | Age group (years) | Men (*n* = 594) | Women (*n* = 646) |
| --- | --- | --- | --- |
|  |  |  |  |
| Phase angle | 65–69 | 5.41 ± 0.58 | 4.78 ± 0.43 |
|  | 70–74 | 5.11 ± 0.57 | 4.54 ± 0.51 |
|  | 75–79 | 4.93 ± 0.57 | 4.30 ± 0.43 |
|  | 80 | 4.47 ± 0.50 | 4.17 ± 0.48 |

Unweighted mean ± standard deviation (SD).
